# Complete Genome Sequence of *Azoarcus* sp. Strain DD4, a Gram-Negative Propanotroph That Degrades 1,4-Dioxane and 1,1-Dichloroethylene

**DOI:** 10.1128/MRA.00775-19

**Published:** 2019-08-15

**Authors:** Daiyong Deng, Fei Li, Lei Ye, Mengyan Li

**Affiliations:** a Department of Chemistry and Environmental Science, New Jersey Institute of Technology, Newark, New Jersey, USA; b HaploX Biotechnology Co., Ltd., Shenzhen, Guangdong, China; University of Maryland School of Medicine

## Abstract

*Azoarcus* sp. strain DD4 can cometabolically degrade 1,4-dioxane and 1,1-dichloroethylene (1,1-DCE) when grown with propane and other substrates. The complete genome sequence of strain DD4 reveals a diverse collection of bacterial monooxygenase genes that may contribute to its versatility in degrading commingled groundwater pollutants.

## ANNOUNCEMENT

Azoarcus sp. strain DD4 is a propanotrophic bacterium isolated from an activated sludge sample collected at a municipal wastewater treatment plant in northern New Jersey (1). Notably, DD4 presents a synchronic degradation ability for 1,4-dioxane and 1,1-dichloroethylene (1,1-DCE) via cometabolism with the induction of propane and some other substrates (1). Like other *Azoarcus* strains, DD4 is also a diazotroph that can assimilate atmospheric nitrogen (1–3). The growth and activity of DD4 can be sustained under a wide variety of aquifer-relevant conditions (1), suggesting that it has potential as an effective inoculum for *in situ* or *ex situ* bioaugmentation to treat the commingled contamination of 1,4-dioxane and 1,1-DCE. Therefore, the whole-genome sequence of DD4 provides insights into the genetic basis of its lifestyle and degradation capabilities, which are valuable to optimize and assess its field applications.

DD4 cells were harvested at the exponential phase after growth in nitrate mineral salts (NMS) medium with propane (0.1% [vol/vol] in the headspace) as the sole carbon and energy source. Total genomic DNA of DD4 was extracted using the MagAttract high-molecular-weight (HMW) DNA kit (Qiagen, Hilden, Germany) according to the manufacturer’s instructions. The extracted DNA was purified with AMPure PB magnetic beads and further used for the library preparation using the combination of the SMRTbell damage repair kit and barcoded adapter complete prep kit (Pacific Biosciences, Menlo Park, CA). The genome of DD4 was sequenced using the PacBio Sequel system, which generated approximately 1.69 Gbp of long-read sequencing data. The average length of raw sequences for sample DD4 is estimated as 2.1 kb, as the final number of raw reads is 802,558. Following the Hierarchical Genome Assembly Process (HGAP), a DD4 genome of high quality and accuracy was assembled using the RS_HGAP_Assembly.3 protocol and polished by Quiver in SMRT Portal v2.3.0 with default parameters (4). For genome component prediction, the GeneMarkS+ program (5) was employed to retrieve the related coding genes. Seven databases were then used for the annotation of gene functions (E value, <1E−5; minimal alignment length percentage, greater than 40%) (6), namely, Gene Ontology (GO) (7), Kyoto Encyclopedia of Genes and Genomes (KEGG) (8), Clusters of Orthologous Groups (COG) (9), non-redundant protein (NR) databases (10), Transporter Classification Database (TCDB) (11), Swiss-Prot, and TrEMBL (12).

There exists one single circular chromosome in DD4 without circular or linear plasmids. The genome size of DD4 is 5,400,077 bp, with a GC content of 66.7%. A total of 5,001 putative genes are annotated, covering approximately 90.1% of the genome. The genome of DD4 contains 57 tRNA genes and 4 rRNA gene clusters (5S, 16S, and 23S). Five gene clusters encoding soluble di-iron monooxygenases (SDIMOs) (13, 14) are found on the chromosome. Based on phylogenetic analysis of the amino acid sequences of their hydroxylase alpha subunits, these five SDIMOs are categorized as two group 1 phenol hydroxylases, one group 2 toluene monooxygenase, one group 3 butane monooxygenase, and one group 5 propane monooxygenase. In addition, genes encoding a copper membrane particulate monooxygenase (15) and a cytochrome P450 CYP153 alkane hydroxylase are identified. One or more of these monooxygenases in DD4 may be responsible for initiating the oxidation of propane, 1,4-dioxane, 1,1-DCE, and other environmental pollutants (1, 16–19).

### Data availability.

The whole-genome sequence of *Azoarcus* sp. strain DD4 has been deposited in GenBank under the accession number CP022958. The raw reads have been deposited in SRA under the accession number PRJNA398544.
